# Melatonin as a Potential Approach to Anxiety Treatment

**DOI:** 10.3390/ijms232416187

**Published:** 2022-12-19

**Authors:** Kristina Repova, Tomas Baka, Kristina Krajcirovicova, Peter Stanko, Silvia Aziriova, Russel J. Reiter, Fedor Simko

**Affiliations:** 1 Institute of Pathophysiology, Faculty of Medicine, Comenius University, Sasinkova 4, 81108 Bratislava, Slovakia; 2 Department of Cell Systems and Anatomy, UT Health San Antonio, Long School of Medicine, San Antonio, TX 78229, USA; 3 3rd Department of Internal Medicine, Faculty of Medicine, Comenius University, 83305 Bratislava, Slovakia; 4 Institute of Experimental Endocrinology, Biomedical Research Center, Slovak Academy of Sciences, 84505 Bratislava, Slovakia

**Keywords:** anxiety, depression, melatonin, sympathetic nervous system, neurohumoral activation, oxidative stress

## Abstract

Anxiety disorders are the most common mental diseases. Anxiety and the associated physical symptoms may disturb social and occupational life and increase the risk of somatic diseases. The pathophysiology of anxiety development is complex and involves alterations in stress hormone production, neurosignaling pathways or free radical production. The various manifestations of anxiety, its complex pathophysiological background and the side effects of available treatments underlie the quest for constantly seeking therapies for these conditions. Melatonin, an indolamine produced in the pineal gland and released into the blood on a nightly basis, has been demonstrated to exert anxiolytic action in animal experiments and different clinical conditions. This hormone influences a number of physiological actions either via specific melatonin receptors or by receptor-independent pleiotropic effects. The underlying pathomechanism of melatonin’s benefit in anxiety may reside in its sympatholytic action, interaction with the renin–angiotensin and glucocorticoid systems, modulation of interneuronal signaling and its extraordinary antioxidant and radical scavenging nature. Of importance, the concentration of this indolamine is significantly higher in cerebrospinal fluid than in the blood. Thus, ensuring sufficient melatonin production by reducing light pollution, which suppresses melatonin levels, may represent an endogenous neuroprotective and anxiolytic treatment. Since melatonin is freely available, economically undemanding and has limited side effects, it may be considered an additional or alternative treatment for various conditions associated with anxiety.

## 1. Introduction

Anxiety disorders are the most common mental diseases. The prevalence of anxiety varies from 2.5–7%, while around 63% of patients are females [1]. Conditions such as anxiety and fear induced by stressors are common and normal in everyday life when they are transient. In contrast, anxiety as a pathologic disorder is marked (out of proportion to the actual threat posed) and persistent and impairs daily functioning [2]. According to the Diagnostic and Statistical Manual of Mental Disorders (DSM-5), generalized anxiety disorder (GAD) is excessive anxiety and worry about various events or activities persisting for at least 6 months, while the individual finds it difficult to control the worry. Anxiety and worry are associated with at least three of the following symptoms: restlessness, feeling keyed up, inadequate fatigue, difficult concentration, irritability, muscle tension, sleep disturbance and autonomic arousal in terms of palpitations, sweating, trembling or dry mouth. Anxiety states or physical symptoms cause impairment in social, occupational or other important areas of functioning. Anxiety disorders may have other manifestations, such as panic disorder (PD) with recurrent, unexpected panic attacks, a specific phobia involving fear, anxiety or avoidance of circumscribed objects or situations or social anxiety disorder (social phobia, SAD) characterized by anxiety in situations when a subject during their activities is exposed to the attention of others [3].

The treatment of anxiety disorders includes various approaches ranging from psychotherapies, such as cognitive behavioral therapy or exposure therapy, neurostimulation strategies, such as transcranial magnetic stimulation, to pharmacotherapies. Examples of approved drugs used in anxiety disorders include selective serotonin reuptake inhibitors (SSRIs), serotonin–norepinephrine reuptake inhibitors (SNRIs) and benzodiazepines. SSRIs and SNRIs are the first-line treatments for PD, GAD and SAD. Some of the adverse effects include jitteriness or agitation, especially at the beginning of the treatment, nausea, restlessness, headache, fatigue, changed appetite or weight, tremor or sweating [4]. Benzodiazepines are still widely recommended for anxiety, and the anxiolytic effect begins soon after intake, without initial agitation or insomnia. However, the use of benzodiazepines is associated with depression of the central nervous system (CNS), resulting in fatigue, dizziness, prolonged reaction time and cognitive dysfunction. Moreover, long-term use of benzodiazepines can lead to addiction or tolerance [5]. The recent intensive research on the pathomechanisms of anxiety diseases opens the door to the development of novel anxiolytic drugs. New serotonergic agents, such as vilazodone or vortioxetine, with more favorable adverse effect profiles are studied in GAD [6] and SAD [7]; however, their role in anxiety treatment is still not established. Their adverse effects include discontinuation due to adverse events or nausea and vomiting [8]. Ketamine, an N-methyl-d-aspartate (NMDA) receptor antagonist, originally developed as an anesthetic, shows rapid and sustained antidepressant effects [9]. There are some studies examining the anxiolytic effect of ketamine in SAD, GAD [4,10] and PTSD [11]. However, its safety still has to be evaluated for its euphoric and dissociative effects and increased incidence of undesirable cardiovascular effects, hepatotoxicity and ulcerative cystitis [12,13]. Another substance studied for its anxiolytic effect is cannabidiol (CBD), which is a negative allosteric modulator at the cannabinoid 1 (CB1) receptor, resulting in the lack of CB1-associated psychotropic effects, and a partial agonism at the serotonin 1A receptor, supposedly mediating its anxiolytic and antipanic effects [13,14]. The benefit of CBD in patients with GAD, SAD, specific phobias and PD is under clinical testing (NCT03549819). It seems that CBD might be a promising anxiolytic treatment, although further research is unavoidable [15]. Diverse neuropeptides play a role in anxiety development. Neuropeptide S, neuropeptide Y or oxytocin act as natural anxiolytics; thus, there is a search for molecules mimicking their effect or enhancing their anxiolytic actions [13]. On the other hand, neuropeptides such as corticotropin-releasing hormone, vasopressin, substance P or cholecystokinin exert opposing effects—enhancing anxiety. The blocking of their functions might be another line of anxiolytic drugs [13]. For detailed information on current and novel anxiety pharmacotherapy, the reviews by Garakani et al. [4] and Sartori and Singewald [13] are recommended.

Since pathologic anxiety negatively influences a broad spectrum of common daily activities, is manifested in various clinical forms and the current therapy is of limited effectiveness and exerts significant side effects, there is a constant, ongoing search for novel approaches for treating anxiety. Although there are some clinical trials testing novel anxiolytics, the results are not convincing [4]. This review will explore the possible beneficial role of melatonin in reducing anxiety. Melatonin seems to interfere with several potential humoral and signaling mechanisms that participate in anxiety disorder development and, due to its favorable safety profile, is discussed as an additional strategy for treatment of this mental disorder.

## 2. Neurohumoral Mechanisms Contributing to Anxiety

### 2.1. The Role of the Sympathetic Nervous System in Anxiety

The stress response is a reaction to physical or psychological stressors that disrupt the homeostasis mediated by an interplay of the sympathetic nervous system (SNS), the hypothalamus–pituitary–adrenal (HPA) axis and the immune system [16]. The SNS acts via the secretion of epinephrine by the adrenal glands, which mediates the rapid response, while the HPA axis is activated after several minutes and starts with the secretion of corticotropin-releasing factor (CRF) from the paraventricular nucleus of the hypothalamus (PVN) [17].

**Figure 1:**
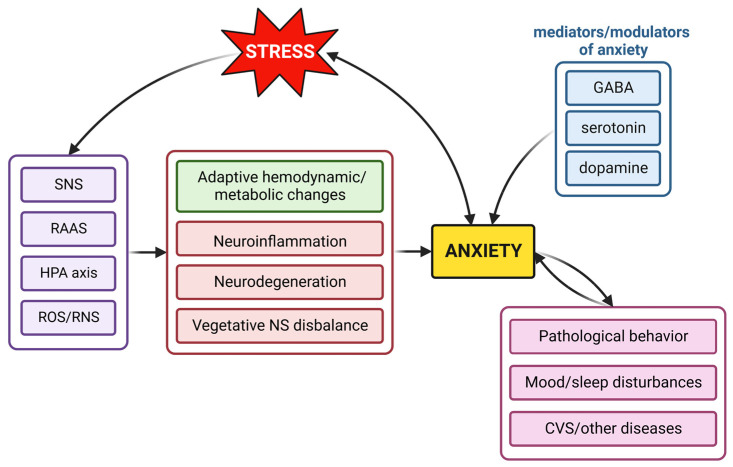
Proposed mechanisms of anxiety development. During the stress reaction, neurohumoral systems including the sympathetic nervous system (SNS), renin–angiotensin–aldosterone system (RAAS) and hypothalamus–pituitary–adrenal (HPA) axis are activated, and free radical formation is enhanced. As a result, aside from adaptive hemodynamic and metabolic changes, inflammatory and degenerative alterations and the disbalance of the vegetative nervous system (NS) occur. The result is the development of anxiety, which, in turn, potentiates or modifies the stress reaction. Established anxiety results in pathological behavior, mood and sleep disturbances and potentially in cardiovascular (CVS) and other systems’ disorders, while each of them or together may further promote anxiety. GABA, γ-aminobutyric acid; ROS, reactive oxygen species; RNS, reactive nitrogen species.

The activated SNS seems to participate significantly in anxiety. The secretion of salivary alpha-amylase (sAA), a digestive enzyme produced by the salivary glands, increases after SNS stimulation [18]. Thus, sAA was proposed to be a surrogate marker of SNS activity [18]. Accordingly, the level of sAA increases in response to acute and chronic stress and in anxiety [19]. In healthy middle-aged women, the urinary excretion of norepinephrine (NE) was found to be increased during anxiety [20]. Patients with GAD showed an elevated plasma NE level [21] and higher sAA levels [22] in comparison to controls. PD patients have reduced NE reuptake maintaining the neural activation [23] as well as higher sAA levels [24]. In posttraumatic stress disorder (PTSD), the circulating levels of NE are increased [25] with an opposite diurnal pattern of sAA secretion [26]. These findings suggest that the autonomic nervous system is dysregulated in different anxiety disorders. Moreover, acute exposure to stressors alters the stress response in anxiety patients. In reaction to acute mental and physiological stress, the muscle sympathetic nerve activity (MSNA) burst amplitude was augmented in both GAD [21] and PTSD [27] patients. PD patients that experienced spontaneous panic attacks after acute mental strain showed elevated epinephrine levels, NE spillover and augmented MSNA amplitude [28] (Figure 1).

Accordingly, the blockade of SNS reduces anxiety levels. After a social defeat in Sprague Dawley rats, pretreatment with propranolol and nadolol increased the time spent in the lit area of a light/dark box (LDB) and in the open arms of an elevated plus maze (EPM) [29] that is considered an anxiolytic-like behavior. In clinical conditions, beta-blockers reduced anxiety symptoms in patients with panic disorder [30], specific phobias [31], social phobias [32] and PTSD [33].

### 2.2. The Hypothalamus–Pituitary–Adrenal Axis in Anxiety

When a stressor is recognized, the PVN of the hypothalamus activates the HPA axis response. First, CRF is released from the PVN, subsequently stimulating the synthesis and release of adrenocorticotropic hormone (ACTH) from the anterior pituitary. ACTH acts on the cortex of the adrenal glands, resulting in glucocorticoid synthesis and secretion [16]. Glucocorticoids bind to the glucocorticoid and the mineralocorticoid receptor. In the brain, they modify the immune, cognitive, metabolic and other systems, altering the physiology and behavior [16]. Cortisol influences its own synthesis and release through the negative feedback mechanisms that regulate HPA axis activity. In chronic stress, the negative feedback may be altered, and along with other modifications, the result is the hyperactivity of the HPA axis and subsequent development of mood and anxiety disorders [34].

In PD patients, the plasma and salivary cortisol [35] and ACTH [36] levels were increased. Elderly individuals with current or past GAD showed upregulated diurnal cortisol secretion [37], and patients with a specific driving phobia experienced increased cortisol levels before, during and after a driving task [38]. Furthermore, the sAA/cortisol ratio seems to be of importance in anxiety disorders. Patients with GAD exhibit greater sAA/cortisol ratios than healthy controls [39]. On the contrary, after a mental arithmetic challenge, patients with GAD exhibited lower sAA/cortisol ratios relative to healthy controls, which is characterized as SNS–HPA asymmetry. It is probable that the suppression of SNS reactivity in GAD is partially mediated by decreased activation of SNS arousal as a result of cortisol-induced SNS suppression [39] (Figure 1).

The blockade of the glucocorticoid effect by mifepristone, a non-selective antagonist to the glucocorticoid receptor, exerts various effects on anxiety [40]. For example, administration of 10 mg/kg of mifepristone for 21 days to male Fischer rats increased latency to explore the open arms of an elevated T-maze, thus indicating anxiety-like behavior [41], while a dose 30 mg/kg for 3 days to male Wistar rats increased the time spent in the central area of the open field test (OFT) and a lower percentage of time spent in the closed arm of the EPM, suggesting an anxiolytic-like effect [42].

### 2.3. The Impact of the Renin–Angiotensin–Aldosterone System in Anxiety

Stress, via increased SNS activity, enhances renin activity, initiating the cascade of the renin–angiotensin–aldosterone system (RAAS). In turn, angiotensin II (Ang II) stimulates catecholamine release from the central locus coeruleus and peripheral adrenal medulla [43]. During the stress response, Ang II type 1 (AT1) receptor expression also increases in the PVN of the hypothalamus, pituitary and peripheral adrenal glands, activating the stress-induced HPA axis and enhancing the sequential production and release of CRF, ACTH and glucocorticoids. In turn, during the stress reaction, glucocorticoids increase AT1 receptor expression in the PVN [44]. Thus, during stress, Ang II stimulates the central and peripheral SNS and all components of the HPA axis, amplifying the stress response.

The RAAS plays a role not only in stress response but also in subsequent anxiety development. The I/D polymorphism of the angiotensin I-converting enzyme (ACE) gene is more common in male patients with panic disorder [45], and primary hyperaldosteronism is associated with GAD [46]. Ang II administration induces anxiety in rodents. The administration of Ang II decreased the time spent in the center of the OFT [47,48] and decreased the time spent in the open arm of the EPM [47,48,49] in mice. Finally, aldosterone administration reduced the frequency of entries, the percentage of time spent in the central area and the number of squares crossed in the central area of the OFT, indicating anxiety-like behavior in rats [50] (Figure 1).

The role of the RAAS is supported by findings that the blockade of the RAAS mediates the anxiolytic effect in humans and animals [51,52]. Patients taking ACE inhibitors or angiotensin receptor blockers (ARBs) more effectively reduced traumatic stress symptoms compared to beta-blockers or calcium channel blockers in patients with PTSD [53]. Losartan decreased the response of the amygdala to stimuli associated with fear [54]. The discontinuation of valsartan induced significant anxiety symptoms that were relieved after restarting valsartan [55]. The inhibition of AT1 receptor neurons in the PVN in mice [56] and AT1aR gene deletion in the PVN in mice [57] increased the percentage of time spent in the open arms of the EPM. The angiotensin-converting enzyme inhibitor captopril increased the time spent in the central square of the OFT and time spent in the open arms of the EPM in doxorubicin-pretreated rats [58]. The AT1 receptor blockade by losartan increased the time spent in the OPF after a forced swim test [59] and the time spent in the open arms of the EPM in mice treated with lipopolysaccharides [60]. In streptozotocin-induced diabetic rats with anxiety-like behavior, the aldosterone receptor antagonist spironolactone exerted an anxiolytic effect [61], and eplerenone reduced anxiety-like behavior related to exploration and risk assessment behavior in Wistar rats [62]. The anxiolytic effect of RAAS blockade may also be mediated by the upregulation of the Ang II type 2 receptors in the brain [63], decreasing the central and peripheral SNS activity [64] and HPA response to stress [64,65], or via amelioration of neuroinflammation [52,60].

### 2.4. Reactive Oxygen and Nitrogen Species in Anxiety Development

High oxygen consumption coupled with modest antioxidant defense predisposes the brain to generate reactive oxygen species (ROS), while brain overexpression of neuronal and inducible nitric oxide synthase (nNOS and iNOS) contributes to reactive nitrogen species (RNS) production. ROS/RNS may damage nucleic acids, proteins and neuronal lipid membranes and thus underlie the altered neuronal function considered in the pathogenesis of various neurodegenerative or neuropsychiatric diseases [66,67].

Several data also indicate a role of oxidative and nitrosative stress in the pathophysiology of anxiety. Hovatta et al. classified six mouse strains by anxiety and found that the brain expression of glutathione reductase 1 and glyoxalase 1—enzymes involved in antioxidant defense mechanisms—was higher in more anxious strains [68]. The overexpression of the antioxidant enzymes is believed to be a response to uncontrolled ROS/RNS production [66]. Moreover, regulation of the expression of genes encoding glutathione reductase 1 and glyoxalase 1 in the cingulated cortex was shown to modulate the anxiety-like behavior of mice [68]. Importantly, anxiety-like behavior in mice was associated with significantly higher intracellular ROS levels in neuronal and glial cells of the cerebellum and hippocampus, neurons of the cerebral cortex as well as peripheral blood cells [69,70].

L-ascorbic acid (vitamin C) is a water-soluble antioxidant that is essential to several mammals, including humans. The high intracellular concentration of vitamin C in neurons suggests its essential role in neuroprotection via its potent antioxidant and free radical scavenging properties [71]. As reviewed by Moritz et al. [72], vitamin C supplementation reduced anxiety indices in rodents [73,74,75,76] and humans [77,78], while reducing the ROS/RNS burden [73,74,75].

Furthermore, oxidative/nitrosative stress induced by buthionine-S,R-sulfoximine (BSO) treatment was associated with increased anxiety-like behavior in both rats and mice [79,80]. In contrast, treatment with the antioxidant tempol attenuated BSO-induced oxidative/nitrosative stress and the anxiety-like behavior [80]. Of note, nNOS and iNOS were shown to be overexpressed in social stress-induced anxiety in the rat brain, and their downregulation had anxiolytic effects [81,82]. Finally, in clinical settings, increased ROS formation in mononuclear cells was associated with anxiety in hypertensive patients [83], and increased levels of antioxidant enzymes in red blood cells and malondialdehyde (marker of lipid peroxidation) in plasma were found in subjects with anxiety disorders, including obsessive–compulsive or panic disorder [84,85] (Figure 1).

### 2.5. The Alterations of Neurotransmitters in Anxiety

Serotonin (5-hydroxytryptamine, 5-HT) is a significant neurotransmitter modulating various physiological functions (e.g., the sleep–wake cycle, emotions, stress reaction) [86]. Serotonergic system dysregulation is linked with anxiety [87]. The majority of serotonergic cells occupy the raphe nuclei, especially the dorsal raphe nucleus (DRN) [88]. Serotonin released from the DRN acting via 5-HT_2C_ receptors within the amygdala was found to enhance fear and anxiety [89]. On the contrary, 5-HT_1A_ receptor stimulation was associated with anxiolysis [90]. Most anxiolytic and antidepressant drugs target the 5-HT system. Broad distribution of the 5-HT neurons in the CNS and the possibility of non-synaptic diffuse neurotransmission and disposal of 5-HT receptors together provide the concept of the complex interaction between serotonin and other neurotransmitter systems [88] (Figure 1).

γ-Aminobutyric acid (GABA) is the main inhibitory neurotransmitter in the CNS of humans and rodents. The GABAergic system is a complex network encompassing several CNS areas, while having multiple reciprocal connections with other neurotransmitter systems [91]. GABAergic neurons represent the second major cell population in the DRN, and 5-HT neurons gain GABAergic inputs. The dysregulation of GABAergic signaling in the DRN was shown to be associated with anxiety [92]. The amygdala is responsible for the creation and the repository of fear memories. Neurons of the amygdala are distinctive for the low firing activity due to a heavy inhibitory tone, stoppable only by a signal indicating threat that activates the downstream defensive circuits via excitatory neurons [93]. Signaling which does not forecast danger is suppressed by interneurons. Pathological impairment of the mentioned suppression may cause maladaptive fear and anxiety [94]. In general, GABA receptor agonists promote anxiolysis, while antagonists exert anxiogenic effects [95] (Figure 1).

Dysfunctional neurotransmitters and receptors in the dopaminergic system in the mesolimbic, mesocortical and nigrostriatal areas interfering with the glutamatergic system in the medial prefrontal cortex are involved in anxiety [96]. Dopamine is released in the amygdala under stressful conditions [97] and has a suppressing effect on inhibition of anxiety by the prefrontal cortex [98], which allows anxiety to be freely expressed [99]. Both types of dopamine receptors, D1 and D2, are involved in modulation of anxiety. D1 receptors in the amygdala are activated in conditioned and unconditioned fear and their blockade has an anxiolytic effect [100]. The role of D2 receptor in anxiety and unconditioned fear is unclear [100] (Figure 1).

## 3. Melatonin: Synthesis, Phylogenetic Considerations, Signaling Pathways and Effects

### 3.1. Melatonin Synthesis, Evolution and Signaling Pathways

**Figure 2:**
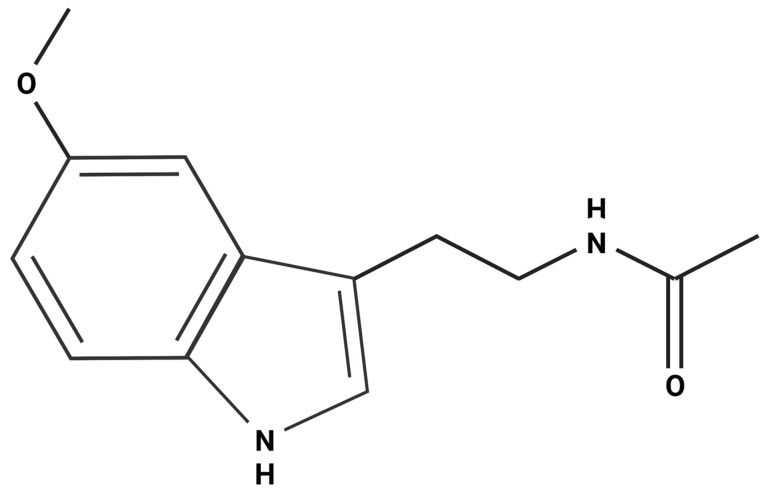
Biochemical structure of melatonin (*N*-acetyl-5-methoxytryptamine).

Melatonin (*N*-acetyl-5-methoxytryptamine) is the principal secretory product of the pineal gland which is a major source of this indolamine [101] (Figure 2). Experimental pinealectomy substantially reduces circulating melatonin levels [102]; however, it does not lead to complete eradication of melatonin from plasma, indicating that the pineal gland is the main but not the exclusive source of melatonin [101,103]. Indeed, a number of extrapineal tissues, including the gastrointestinal tract [104], immune system [105], kidney, heart and others, were shown to produce melatonin [103]. Melatonin in the extrapineal tissues acts via intracrine, autocrine and paracrine manners [105] and is poorly released into circulation; therefore, the circulating indolamine depends mainly on the pineal secretion [106]. Melatonin was originally discovered as a pineal gland secretory product in vertebrates, but its production was later shown in all animals and also plants, fungi and unicellular organisms [103,107].

Melatonin is an ancient molecule associated with the phylogenetic development from unicellular organisms to multiorgan living systems without any structural modification over millions of years [107]. Primitive photosynthetic bacteria, where melatonin probably evolved as a protection against oxidative stress, were theoretically phagocytized by early eukaryotes and developed into mitochondria and chloroplasts. During evolution, via the distribution of mitochondria, melatonin was subsequently spread to all multicellular organisms with new sites of generation and functional implications [103,107]. Although the melatonin synthetic pathway is slightly different in plants and animals, the stepwise enzymatic conversion from the amino acid tryptophan to serotonin and melatonin represents the principal metabolic route [107].

**Figure 3:**
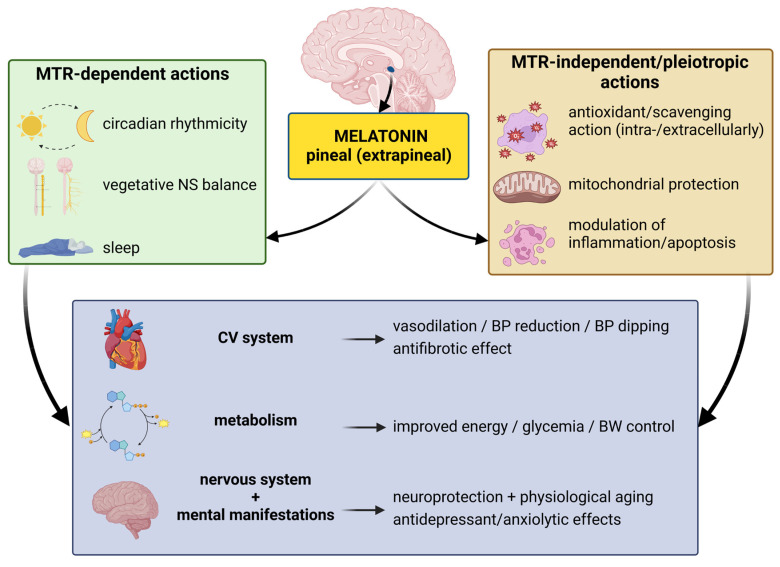
Receptor-dependent and receptor-independent effects of melatonin. MTR, melatonin receptor; CV system, cardiovascular system; BP, blood pressure; BW, body weight.

Melatonin is an unusually multitasking molecule that participates in an uncountable number of physiological and pathological actions [108,109,110]. Although the modulation of circadian rhythms is considered to be a principal role of melatonin, this indolamine exerts protection in various organs and systems. In the cardiovascular system, it participates in blood pressure (BP) regulation and BP decline during the night and exerts antifibrotic effects in the left ventricle [111,112]. Melatonin has also been shown to improve glucose and lipid metabolism, modulate body weight and energy metabolism, attenuate neurodegenerative processes and depressive and anxiety behavior and expresses anticancer actions [103,109,113]. This indolamine exerts its effects either via specific membrane receptors, cytoplasm or nucleus-bound proteins or via receptor-independent biological actions (Figure 3). Two types of melatonin-specific G-protein-coupled membrane-bound receptors were identified as MT1 and MT2 [114]. The action via the Gi protein is mediated by a reduction of cyclic adenosine monophosphate, with attenuation of protein kinase activity, and via the Gq protein that is followed by phospholipase and protein kinase C activation. The subsequent signaling may result in an increased level of intracellular calcium and nitric oxide (NO) formation [115]. Other melatonin-binding sites with obscure biological impacts involve MT3/quinone reductase (QR2) in membranes [116], nuclear retinoid orphan receptors/Z receptors (RORs/RZRs) [117] and intracellular proteins, e.g., calmodulin or tubulin [118].

### 3.2. Pleiotropic Receptor-Independent Actions

**Figure 4:**
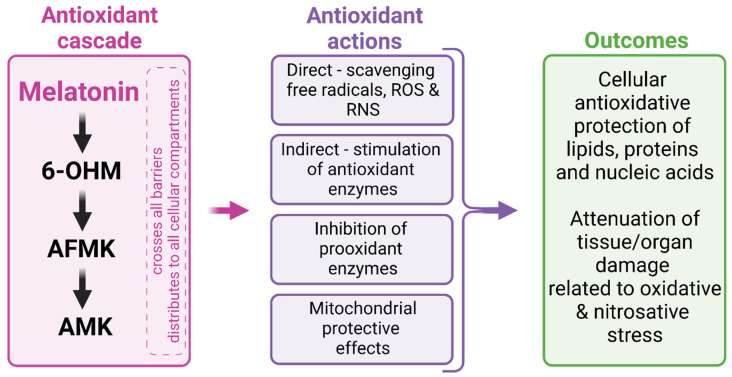
Mechanisms of melatonin’s antioxidant actions. 6-OHM, 6-hydroxymelatonin; AFMK, N1-acetyl-N2-formyl-5-methoxykynuramine; AMK, N1-acetyl-5-metoxykynuramine; RNS, reactive nitrogen species; ROS, reactive oxygen species.

Potentially, many biological effects of melatonin are receptor-independent actions (Figure 3). Melatonin is one of the most effective means of limiting the harm of free radical stress both extra- and intracellularly, either by preventing free radical generation or, once produced, by neutralizing them. First, melatonin can directly inactivate free radicals by donating one or more electrons [119,120]. Furthermore, melatonin enhances the expression and activity of antioxidant enzymes such as catalase, superoxide dismutase or glutathione peroxidase [121] and enzymes required for glutathione synthesis and recycling, including glutathione synthetase, glutathione reductase and gamma-glutamyl transpeptidase [122]. Finally, melatonin inhibits prooxidant enzymes, particularly nitric oxide synthase and lipoxygenase [123]. Importantly, melatonin was shown to exert synergistic antioxidative actions with other common antioxidants, such as vitamin C, vitamin E or glutathione [124], while melatonin was even more effective in prevention of oxidative DNA damage than vitamin C, alpha lipoic acid and resveratrol [125]. Additionally, electron donation is the basis for the production of the melatonin derivates, such as 6-hydroxymelatonin (6-OHM), N1-acetyl-N2-formyl-5-methoxykynuramine (AFMK) and N1-acetyl-5-methoxykynuramine (AMK), that have been defined as melatonin’s antioxidant cascade [119]. Interestingly, AFMK and AMK exert excellent ·OH radical scavenging activity, but seem to be ineffective in neutralization of ·OOH. AMK seems to be a more potent scavenger of ·OH than melatonin, while melatonin is superior in neutralizing ·OOH compared to AMK [126]. Of note, 3-hydroxymelatonin (3-OHM) was shown to be a more effective copper chelator than AFMK, AMK or melatonin [127]. Thus, melatonin and its derivates seem to be complementary players in different conditions [126] (Figure 4).

Melatonin has been recognized as a modulator of the immune system. A number of inflammatory cells have been shown to produce melatonin, including monocytes, T-lymphocytes and mast cells [105,128]. Its inflammation-modulatory action seems to depend on the phase of the inflammatory reaction, which acts as a stimulant under basal conditions or as an anti-inflammatory factor in the case of excessive immune response [105,129]. Although melatonin is not directly viricidal, it can attenuate tissue damage due to exaggerated immune response to a microbial infection and has been suggested as a potential protective means in COVID-19 treatment [130,131,132,133]. Similarly, in non-inflammatory apoptotic cell death, melatonin may exert either an anti- or proapoptotic impact depending on the particular pathological conditions [123,134].

Melatonin has significant antioxidative implications in mitochondria [135,136,137]. This indolamine may not only be taken up from circulation by crossing cellular and mitochondrial membranes, but it also seems to be locally synthesized in a pyruvate/serotonin/*N*-acetylserotonin/melatonin pathway [137]. Melatonin exerts protection at several levels. Besides the direct scavenging action, melatonin stimulates the effect of the major mitochondrial antioxidant enzyme superoxide dismutase 2 via sirtuin 3 activation. This reaction preserves the efficiency of the electron transport chain, thus supporting adenosine triphosphate (ATP) generation and improving cellular energy metabolism. Furthermore, melatonin stimulates the transport of mitochondria from healthy cells to damaged cells through tunneling nanotubules, thus reducing apoptotic cell destruction [137] (Figure 3 and Figure 4).

### 3.3. Melatonin and Circadian Rhythmicity

The principal receptor-dependent melatonin action is to coordinate the circadian rhythms of various physiological functions in relation to the rotation of Earth on its axis during one day [106,113,138]. Specific melatonin receptors express the high density in brain structures, such as the suprachiasmatic nucleus (SCN) [139], the adenohypophysis [140] or the PVN [141], which coordinate circadian rhythm variations in close cooperation with melatonin. Although each organ, system, tissue or even each cell supposedly exerts circadian oscillations, the core regulator and coordination of biological rhythms is located in the hypothalamic SCN, which generates a rhythmic oscillation of 24 h. The coordination of SCN with the periphery is maintained by the nervous system and by humoral factors, whose leader is circulating melatonin of the pineal gland [138]. This indolamine is supposed to modify and coordinate both SCN-controlled and SCN-independent oscillators, the expression of potentially rhythmic genes and the production of rhythmically secreted hormones, while all these levels work in mutual cooperation [106,123]. The stimulus for melatonin synthesis and secretion is darkness, and light inhibits its production. Thus, artificial light production at night leads to a shift in melatonin production with potential health disturbances from chronodisruption [113,142].

### 3.4. Anxiolytic Effect of Melatonin

Several animal [143,144,145,146,147,148,149,150,151,152] and human [153,154,155,156,157,158,159,160,161] studies have shown that melatonin administration exerts an anxiolytic effect. In experimental studies, various paradigms are used to test the anxiety-related behavior of animals. Naturally, rodents prefer dark and closed areas that offer protection and avoid lit and open spaces. Briefly, increased time spent in dark and closed areas is considered to be an anxiety-like behavior. Examples of anxiety behavioral assays include an open field test, an elevated plus maze, a light–dark box or a novelty-suppressed feeding (NSF) [162].

The surgical removal of the pineal gland, or pinealectomy, compromises the rhythmicity of melatonin release and reduces plasma melatonin concentration [101]. The melatonin deficit induced by pinealectomy reduced the entry to and the percentage of time spent in the open arm of the EPM in adult male rats [163] and increased the cumulative burying behavior and burying behavior latency in a burying behavior test [146], indicating anxiety-like behavior.

Melatonin administration increased the activity in the central area of the OFT in healthy rats [143]. Melatonin increased the time spent in the open arms of the EPM in healthy rats [144,145], pinealectomized rats [146], diazinon-treated rats [147] and hypertensive rats with an upregulated renin–angiotensin system [148]. After traumatic brain injury in rats, melatonin increased the time spent in the center of the OFT, the percentage of open arm entries and the percentage of open arm times in the EPM [149]. In mice, melatonin increased the time spent in the lit box as well as the number of transitions between the two compartments of the LDB [150,151] and amplified the percentage of time spent in the open arms of the EPM [152]. All these results support the role of melatonin in attenuating anxiety-related behavior.

In humans, melatonin has been tested as a premedication to prevent preoperative and postoperative anxiety, as an adjunct to anesthetic drugs, as an analgesic and for the prevention of postoperative delirium. Clinical studies showed that melatonin given as premedication could reduce preoperative anxiety in patients undergoing abdominal hysterectomy [153], hand surgery during intravenous regional anesthesia [154] or other elective surgery [155] and is equally effective as the standard anxiolytic treatment with benzodiazepines [155,156,157]. In children undergoing minor elective surgery, melatonin, equally to midazolam, alleviated preoperative separation anxiety and anxiety associated with the introduction of the anesthesia mask [158,159].

The postoperative anxiety following laminectomy [160] and colorectal surgeries [161] was reduced after melatonin premedication. Melatonin diminished the postoperative emergence delirium in children undergoing elective infraumbilical surgery [164] and after sevoflurane anesthesia in children undergoing elective ambulatory procedures [165]. Moreover, some clinical studies observed melatonin’s superiority to midazolam in reducing the incidence of postoperative excitement [158], emergence delirium [165] and sleep disturbances [158]. Melatonin also reduced postoperative pain in patients with colorectal surgery [158,161] and abdominal hysterectomy [153], while decreasing morphine consumption [153] as well as in children undergoing elective infraumbilical surgery [164]. Moreover, melatonin is associated with faster recovery, lower incidence of excitement and a lower incidence of sleep disturbance postoperatively [158].

In summary, melatonin reduces both preoperative and postoperative anxiety, while working as effectively as benzodiazepines [157]. While benzodiazepines impair psychomotor and cognitive function after anesthesia, melatonin does not [155]. The randomized, placebo-controlled trials identified only a few mild adverse effects after melatonin treatment, such as daytime sleepiness and other sleep-related adverse effects, headache, dizziness and hypothermia [166]. Only a few serious or clinically significant adverse events were reported, such as agitation, fatigue, mood swings, nightmares, skin irritation and palpitations. Almost all adverse effects resolved spontaneously within a few days or immediately after melatonin discontinuation, while the rate of adverse effects was not markedly different from that for placebo. Importantly, no life-threatening adverse effects of melatonin or those of major clinical significance were identified [166]. Of note, melatonin was effective in counteracting antipsychotic drug-induced metabolic side effects. Melatonin reduced BP, weight gain or cholesterol levels induced by antipsychotic treatment [167]. In conclusion, melatonin seems to be an attractive alternative to benzodiazepines in relieving the anxiety associated with surgical procedures in children and adults while being considered a safe and well-tolerated drug.

The mechanisms of melatonin’s observed anxiolytic effect are not entirely understood. Melatonin’s anxiolytic effects might be associated with direct or indirect mechanisms. Direct ones are related to melatonin receptors in the brain and indirect ones to melatonin’s ability to modulate various neurohumoral systems, including the SNS, the RAAS, glucocorticoids and neurotransmitters, thus potentially interfering with the stress reaction and circadian rhythms, and modifying oxidative and nitrosative stress and inflammation.

MT1 and MT2 receptors have been shown to play a role in anxiety-like responses. These melatonin receptors were identified in brain areas involved in fear processing, such as the amygdala, hippocampus and prefrontal cortex [168]. The melatonin MT1 receptor knockout mice took a longer time to eat in the novel environment in the NSF test [169,170] and buried more marbles in the marble-burying test [170]. Female mice with a genetic deletion of the MT2 receptor increased thigmotaxis and reduced time spent in the center of the cage [171]. MT2 receptor knockout mice displayed a longer latency to feed in the NSF test [87,170] and shorter latency to enter the dark compartment of the LDB [87]. Transgenic mice with genetic deletion of the MT1/MT2 receptors showed increased latency to feed in the NSF test [170]. In contrast, MT1/MT2 receptor stimulation by S23478 reduced the duration of immobility and ultrasonic vocalization in defeated rats and increased wall-climbing and rearing in anticipating a social defeat test [172]. The selective stimulation of MT2 receptors by UCM765 increased the time spent in the open arms of the EPM and decreased the latency to eat in the NSF test in rats [145]. These data indicate the potential anxiolytic-like behavior mediated by MT1 and MT2 receptors.

The following sections discuss the possible indirect pathways of melatonin’s anxiolytic effect.

#### 3.4.1. Melatonin–Sympathetic Nervous System Interactions

**Figure 5:**
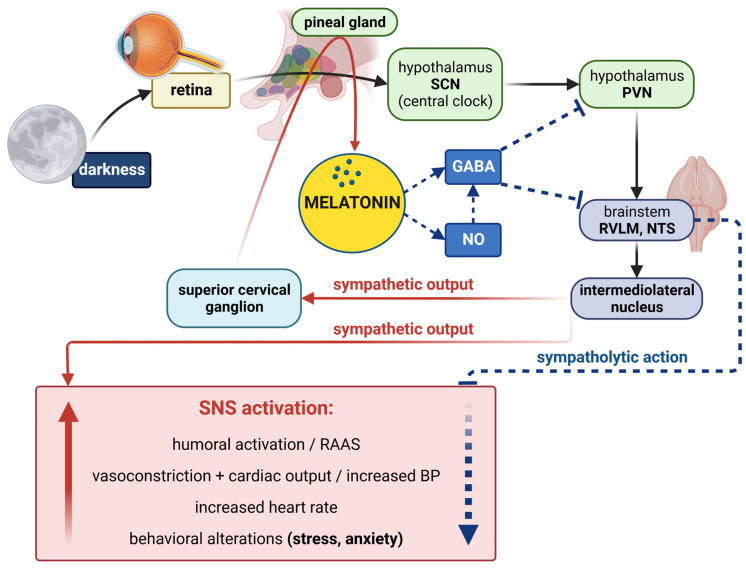
Interaction of the sympathetic nervous system (SNS) with melatonin. Darkness (the absence of light impulses) is the driver of melatonin synthesis and release. The retinohypothalamic tract leads from melanopsin-containing retinal neurons to the suprachiasmatic nucleus (SCN), which generates rhythmic oscillations. These impulses pass via the neurons of the paraventricular nucleus (PVN) of the hypothalamus and via the rostral ventrolateral medulla (RVLM) and nucleus of the solitary tract (NTS) to the upper thoracic intermediolateral cell column, which innervates the superior sympathetic ganglion, generating a sympathetic tone. The sympathetic impulses enhance the peripheral vascular tone and cardiac contractility with a blood pressure (BP) increase, and they activate additional humoral systems and induce behavioral changes. At the same time, the sympathetic impulses from the intermediolateral nucleus project to the superior cervical ganglia from where postganglionic fibers enter the pineal gland to stimulate melatonin production via beta and alpha-1 adrenoceptor activation by norepinephrine. Furthermore, melatonin released from the pineal gland can presumably exert a negative feedback effect on the central sympathetic system via enhancing γ-aminobutyric acid (GABA)ergic signaling, which is involved in the inhibition of PVN, RVLM and NTS. Melatonin also enhances nitric oxide (NO) availability, which potentiates the GABAergic inhibitory effects in the PVN and RVLM. Red arrows represent the sympathetic impulses and blue arrows represent the sympatholytic impulses.

Production and effects of melatonin are interrelated with the sympathetic nervous system on various levels. The retinohypothalamic tract leads from melanopsin-containing retinal neurons to the SCN, generating rhythmic oscillations. The neurons of the PVN in the hypothalamus pass directly or after interpolation in the rostral ventrolateral medulla (RVLM) to the medullar intermediolateral cell column, which innervates sympathetic ganglia generating a sympathetic tone enhancing the peripheral vascular tone and cardiac contractility with a blood pressure increase [173]. Simultaneously, the sympathetic impulses from the intermediolateral column project to the superior cervical ganglia by a preganglionic sympathetic axon to stimulate melatonin production by the pineal gland via beta and alpha-1 adrenoceptor activation [173,174] (Figure 5).

The constant excitatory output of the PVN is intermittently inhibited by GABAergic innervation from the SCN [175]. Melatonin release from the pineal gland, which is triggered by the absence of light impulses [113], could modulate the central sympathetic system on several levels: melatonin may bind to its receptors in the SCN or area postrema, both with a high melatonin receptor density [139,173]. Moreover, melatonin enhances GABAergic signaling, which is involved in the inhibition of PVN by the SCN and in inhibition of the RVLM [176]. Additionally, melatonin enhances NO availability, which was shown to potentiate the GABAergic inhibitory effects in the PVN and RVLM [177,178]. Indeed, in L-NG-nitro arginine methyl ester (L-NAME)-induced hypertension, peak night-time pineal melatonin concentration in the pineal gland was higher in L-NAME-treated rats than in controls, supposedly by the L-NAME-decreased inhibitory effect of NO on melatonin production in the pineal gland [179]. It seems that melatonin may have a negative feedback effect on the SNS by GABAergic inhibitory signaling on the PVN via the SCN, and NO may potentiate this action. The endogenous melatonin may thus represent a counterregulatory mechanism against excessive sympathetic stimulation [173,174,180] (Figure 5).

The melatoninergic sympatholytic effects have been confirmed in a number of experiments. In spontaneously hypertensive rats (SHRs), acute melatonin administration reduced BP and norepinephrine serum levels [181]. The antihypertensive effect of regular melatonin treatment was associated with reduced plasma norepinephrine concentration and a lower number of beta receptors in the heart [182] and improved baroreflex responses [183]. In neurogenic hypertension induced by clipping one renal artery, melatonin reduced BP, attenuated sympathoexcitation to the ischemic kidney and improved baroreflex control of the heart rate (HR) along with ROS reduction in the brainstem regions regulating BP [184]. In clinical conditions, melatonin attenuated cardiac remodeling and brain–heart sympathetic hyperactivation after myocardial infarction [185] and improved autonomic control in pinealectomized patients [186]. Heart rate is under tight control of the SNS, and an elevated heart rate reliably reflects the dominance of the sympathetic over the parasympathetic nervous system tone. Melatonin’s ability to reduce HR both in animals and humans [187] indicates the sympatholytic nature of this indolamine and underlies the potential protection by melatonin not only in cardiovascular pathologies but also in mood disturbances related to overt sympathetic stimulation, such as anxiety (Figure 5).

#### 3.4.2. Potential Interference of Melatonin with the Renin–Angiotensin–Aldosterone System

The available data indicate that the melatoninergic system may interact with the RAAS:

(1) Angiotensin II and melatonin seem to have opposite roles in cardiovascular pathophysiology. While Ang II increases BP and pathologic remodeling of a hypertensive and failing heart [188], melatonin has been shown to reduce BP in hypertensive patients [111,174,189,190] and in a number of animal models of hypertension [101,112,173,191,192] and to exert antifibrotic effects in hypertensive [101,193,194,195,196,197] and failing heart [198] and in kidney [52,199], similar to the ACE inhibitor captopril [52,193,194,195,196,199].

(2) In patients with non-dipping BP, the reduced excretion of 6-sulfatoxymelatonin in the urine was observed [200]. The chronic subcutaneous infusion of a subpressor dose of Ang II to rats induced a shift in circadian BP rhythm in terms of induction of non-dipping hypertension, as also seen in patients [201]. Tissue angiotensin was earlier detected in the pinealocytes of the pineal gland and locally synthesized angiotensin modified melatonin synthesis in the pineal [202]. Recently, insulin-regulated aminopeptidase (IRAP) in the pineal gland has been detected as a receptor target for Ang IV. Ang IV increased the norepinephrine-induced melatonin synthesis in pinealocytes to a similar degree as Ang II [203]. It was suggested that in rats with Ang II-induced non-dipping BP, peripheral RAAS may interact with the brain RAAS. Ang II produced by glial cells may act on AT1b receptors on pinealocytes, thus stimulating tryptophan hydroxylase, the rate-limiting enzyme of melatonin synthesis. BP alteration was supposedly induced by chronodisruption in BP regulation either via Ang II or through melatonin-induced shift of clock gene expression in the SCN or in the circadian clock of several cardiovascular organs [204].

(3) In 53 patients with chronic kidney disease (CKD), impaired night-time excretion of melatonin metabolite urinary 6-sulfatoxymelatonin tightly correlated with altered urinary excretion of angiotensinogen [205], previously detected as a marker of renal renin–angiotensin system activation and renal damage in CKD patients [206]. Moreover, in a 5/6 nephrectomy rat model of progressive CKD characterized by stimulation of the intrarenal RAAS in terms of enhanced level of angiotensinogen, Ang II and AT1 receptor density, melatonin attenuated intrarenal RAAS activation and renal injury via its antioxidant effect [207]. Melatonin-induced attenuation of the mutual RAAS and free radical stress activation has been suggested to ameliorate CKD progression by this indolamine [208]. On the other hand, in L-NAME hypertension, melatonin reduced BP and prevented fibrotic remodeling of the left ventricle without any effect of the renin–angiotensin system and increased serum aldosterone level [209]. Thus, melatonin might exert a different impact on circulatory and tissue RAAS.

These prevailing protective effects of melatonin in relation to the RAAS may supposedly participate in the anxiolytic impacts of melatonin.

#### 3.4.3. Melatonin vs. Glucocorticoids in Anxiety

Melatonin’s blunting effect on glucocorticoid actions was suggested to be associated with its putative anxiolytic properties. In fact, melatonin was shown to reduce density, affinity or nuclear translocation of glucocorticoid receptors in different tissues, including the brain [210,211]. Furthermore, melatonin treatment counteracted glucocorticoid-induced dysregulation of the HPA axis in rats [212] and protected rat hippocampus from glucocorticoid-induced neurotoxicity [213]. Finally, melatonin suppressed anxiety-like behavior and reduced serum corticosterone level in both chronic immobilization stress in rats [214] and sleep deprivation in mice [215]. In patients with fibromyalgia, melatonin supplementation was associated with decreased urinary cortisol level and anxiety, as assessed by state–trait anxiety test [216].

#### 3.4.4. Melatonin Interaction with Oxidative and Nitrosative Stress in Anxiety

Melatonin and its derivates are considered to be potent free radical scavengers and broad-spectrum antioxidants [135,217]. Moreover, melatonin was shown to modulate NO synthase expression and activity in different tissues, including the brain [218].

Several studies showed that melatonin supplementation reduces oxidative/nitrosative stress, improves antioxidant defense and reduces lipid peroxidation markers in health and disease [219]. Indeed, a meta-analysis of 12 randomized control trials encompassing 521 unhealthy subjects showed that melatonin supplementation was associated with an increase in total antioxidant capacity, elevated activities of glutathione, superoxide dismutase and glutathione peroxidase and a reduction in malondialdehyde (a marker of lipid peroxidation) levels [220]. Interestingly, melatonin’s antioxidant properties were shown to be associated with its putative anxiolytic effects. In fact, melatonin supplementation prevented sleep deprivation-induced anxiety-like behavior in mice, associated with reduced oxidative stress, as determined by increased levels of superoxide dismutase and depressed levels of malondialdehyde in amygdala and serum [221]. The attenuation of sleep deprivation-induced anxiety-like behavior and oxidative stress was also associated with amelioration of neuroinflammation, apoptosis and autophagy [215]. In rats with streptozotocin-induced diabetes, melatonin treatment ameliorated anxiety-like behavior and reversed the increase in malondialdehyde levels and the decrease in reduced glutathione level in both the hippocampus and prefrontal cortex [222].

#### 3.4.5. Melatonin-Induced Modifications of Neurotransmission in Anxiety

DRN serotonergic neuronal activity shows variations during light–dark periods. Since MT1 receptors are present on DRN serotonergic neurons and MT2 receptors in other neuronal populations projecting to serotonergic neurons [223], melatonin is believed to play a local modulatory role. Deterioration of the light–dark cycling activity of 5-HT neurons was associated with anxiety in MT1^−/−^ [224] as well as in MT2^−/−^ [87,171] knockout mice. Modulation of the DRN serotonergic neurons by MT2 receptors was demonstrated by studies with selective MT2 receptor ligands [225]. The synergistic interaction between melatonin and 5-HT2C receptors may induce therapeutic effects in various psychiatric disorders [226].

Effects of melatonin involve GABAergic signaling facilitation via GABA receptor stimulation [176,227]. The interplay between the melatonergic and GABAergic systems, underlying some neuropsychogenic effects of melatonin, such as hypnotic activity, appears to be mediated via the GABAA receptor and can be blocked with GABAergic antagonists [227]. Some of these melatonin-induced modifications of neurosignaling pathways might contribute to its anxiolytic action.

**Figure 6:**
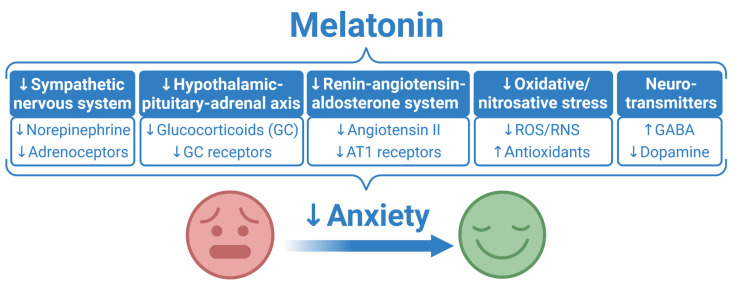
The proposed mechanisms contributing to anxiolytic effect of melatonin. AT1 receptors, angiotensin II type 1 receptors; GABA, γ-aminobutyric acid; GC, glucocorticoids; RNS, reactive nitrogen species; ROS, reactive oxygen species.

Dopamine release appears predominantly during the day and seems to be a counterpart to melatonin released during the night [228]. Through α-D4 and β1-D4 receptor heteromers, dopamine inhibits adrenergic receptor signaling and blocks the synthesis of melatonin induced by adrenergic receptor ligands [229]. Additionally, increased dopamine in the synaptic cleft of the mesolimbic dopaminergic system induced by stress may result in anxiety [96]. On the other hand, various in vitro and in vivo studies have shown that melatonin inhibits dopamine release in hypothalamic areas, ventral hippocampus and striatum [147,230,231]. Thus, melatonin and dopamine seem to represent a reciprocal pair mutually inhibiting the synthesis of each other [232]. Since dopamine seems to exert mostly anxiogenic action [96], melatonin´s inhibitory effect on the dopaminergic system might contribute to the anxiolytic potential of this indolamine (Figure 6).

## 4. Conclusions

Anxiety disorders are the most common mental diseases. They afflict a large proportion of the population and may disturb social and occupational life and various areas of functioning. Moreover, it is generally accepted that anxiety and cardiovascular diseases [52] or other somatic alterations exhibit a bidirectional relation, stimulating or potentiating one another. The pathophysiology of anxiety development is complex and involves alterations in stress hormone production, neurosignaling pathways and free radical production. The various manifestations of anxiety, its complex pathophysiological background and the side effects of available treatment underlie the fact that novel or alternative therapeutic means are constantly being sought. Melatonin, an indolamine produced predominantly in the pineal gland, was demonstrated to exert anxiolytic action in animal experiments and diverse clinical conditions. Melatonin influences many physiologic actions either via specific melatonin receptors or by receptor-independent pleiotropic effects. The underlying pathomechanism of melatonin’s benefit in anxiety may reside in its sympatholytic action, inhibition of the renin–angiotensin and glucocorticoid system, modulation of GABA–serotonergic signaling and its extraordinary antioxidant and scavenging nature (Figure 6). Pineal melatonin is also directly released into the cerebrospinal fluid (CSF), where the concentration of this indolamine is much higher in CSF than in the blood. This enables melatonin to function as a potent antioxidant and anti-inflammatory agent after it diffuses into neural tissue [233], thus potentially participating in the normalization of stress reaction and anxiolysis. Ensuring sufficient melatonin production by reducing light pollution at night and preserving a regular sleep regime may represent an endogenous anxiolytic system. Since melatonin is freely available, economically undemanding and has limited side effects, it may be considered an additional or alternative treatment for different conditions associated with anxiety.

## Data Availability

Not applicable.

## Abbreviations

3-OHM	3-hydroxymelatonin
5-HT	5-hydroxytryptamine
6-OHM	6-hydroxymelatonin
ACE	angiotensin I-converting enzyme
ACTH	adrenocorticotropic hormone
AFMK	N1-acetyl-N2-formyl-5-methoxykynuramine
AMK	N1-acetyl-5-methoxykynuramine
Ang II	angiotensin II
ARBs	angiotensin receptor blockers
AT1 receptor	angiotensin II type 1 receptor
ATP	adenosine triphosphate
BP	blood pressure
BSO	buthionine-S,R-sulfoximine
CB1 receptor	cannabinoid 1 receptor
CBD	cannabidiol
CKD	chronic kidney disease
CNS	central nervous system
CRF	corticotropin-releasing factor
CSF	cerebrospinal fluid
DRN	dorsal raphe nucleus
DSM	Diagnostic and Statistical Manual of Mental Disorders
EPM	elevated plus maze
GABA	γ-aminobutyric acid
GAD	generalized anxiety disorder
HPA axis	hypothalamus–pituitary–adrenal axis
HR	heart rate
iNOS	inducible nitric oxide synthase
LDB	light/dark box
L-NAME	L-NG-nitro arginine methyl ester
MSNA	muscle sympathetic nerve activity
MT1 receptor	melatonin receptor type 1
MT2 receptor	melatonin receptor type 2
NE	norepinephrine
nNOS	neuronal nitric oxide synthase
NO	nitric oxide
NSF	novelty-suppressed feeding
OFT	open field test
PD	panic disorder
PTSD	posttraumatic stress disorder
PVN	paraventricular nucleus
QR	quinone reductase
RAAS	renin–angiotensin–aldosterone system
RNS	reactive nitrogen species
ROR	retinoid orphan receptors
ROS	reactive oxygen species
RVLM	rostral ventrolateral medulla
RZR	retinoid Z receptors
sAA	salivary alpha-amylase
SAD	social anxiety disorder
SCN	suprachiasmatic nucleus
SHR	spontaneously hypertensive rat
SNRIs	serotonin norepinephrine reuptake inhibitors
SNS	sympathetic nervous system
SSRIs	selective serotonin reuptake inhibitors
